# Extracellular vesicles as a new frontier of diagnostic biomarkers in osteosarcoma diseases: a bibliometric and visualized study

**DOI:** 10.3389/fonc.2024.1359807

**Published:** 2024-03-04

**Authors:** Yanhong Pei, Yu Guo, Wei Wang, Boyang Wang, Fanwei Zeng, Qianyu Shi, Jiuhui Xu, Lei Guo, Chaowei Ding, Xiangpang Xie, Tingting Ren, Wei Guo

**Affiliations:** ^1^ Musculoskeletal Tumor Center, Peking University People’s Hospital, Beijing, China; ^2^ Beijing Key Laboratory of Musculoskeletal Tumor, Peking University People's Hospital, Beijing, China; ^3^ Department of Hepatobiliary and Pancreatic Surgery, The Affiliated Cangnan Hospital of Wenzhou Medical University, Cangnan, Zhejiang, China

**Keywords:** osteosarcoma, exosome, biomarker, bibliometric analysis, liquid biopsy

## Abstract

The use of liquid biopsy in cancer research has grown exponentially, offering potential for early detection, treatment stratification, and monitoring residual disease and recurrence. Exosomes, released by cancer cells, contain tumor-derived materials and are stable in biofluids, making them valuable biomarkers for clinical evaluation. Bibliometric research on osteosarcoma (OS) and exosome-derived diagnostic biomarkers is scarce. Therefore, we aimed to conduct a bibliometric evaluation of studies on OS and exosome-derived biomarkers. Using the Web of Science Core Collection database, Microsoft Excel, the R “Bibliometrix” package, CiteSpace, and VOSviewer software, quantitative analyses of the country, author, annual publications, journals, institutions, and keywords of studies on exosome-derived biomarkers for OS from 1995 to 2023 were performed. High-quality records (average citation rate ≥ 10/year) were filtered. The corresponding authors were mainly from China, the USA, Australia, and Canada. The University of Kansas Medical Center, National Cancer Center, Japan, and University of Kansas were major institutions, with limited cooperation reported by the University of Kansas Medical Center. Keyword analysis revealed a shift from cancer progression to mesenchymal stem cells, exosome expression, biogenesis, and prognostic biomarkers. Qualitative analysis highlighted exosome cargo, including miRNAs, circRNAs, lncRNAs, and proteins, as potential diagnostic OS biomarkers. This research emphasizes the rapid enhancement of exosomes as a diagnostic frontier, offering guidance for the clinical application of exosome-based liquid biopsy in OS, contributing to the evolving landscape of cancer diagnosis.

## 1 Introduction

Osteosarcoma (OS), the most prevalent primary malignant bone cancer in children, adolescents, and young people, is a highly aggressive type of tumor characterized by osteoblast differentiation and malignant osteoid production (1). OS not only substantially impacts the physical and mental well-being of individuals but also constitutes a massive socioeconomic burden. Worldwide epidemiological information has revealed that the prevalence of OS is increasing, and the annual rate of new patients is up to 0.8 – 1.1 per 100,000 people (aged 15 – 19 years) (2). Hence, an early diagnosis is essential to improve the prognosis of patients with OS.

The current diagnostic techniques mainly include imaging examinations (X-rays, magnetic resonance imaging, and computed tomography [CT]), and liquid biopsy is considered the standard for diagnosing OS (2, 3). Liquid biopsy comprises collecting circulating tumor DNA (ctDNA), circulating tumor cells (CTCs), cancer-derived exosomes, and other extracellular vesicles (EVs) (4, 5). Although ctDNA and CTCs are mostly used to assess tumor tissue histology, exosomes and other EVs are considered powerful and complementary application platforms. Exosomes, which are robust messengers of tumor and/or normal cell communication networks, contain vast amounts of information that reveal the state of cancer progression, serving as promising biomarkers (6).

Bibliometrics is a multisystem subject that incorporates philology, statistics, and mathematics, among other subjects. The bibliometric analysis method, an emerging in-depth quantitative and qualitative approach, has been widely utilized to visualize the published literature and provide a clearer understanding of recent scientific research (7, 8). Bibliometric assessment mainly focuses on the collaboration networks of authors, institutions, and countries and the interrelation of individual journals and institutions in this field (9). Currently, bibliometrics are broadly used in many subjects, including neurological system (10), digestive system (11), and cancer (12) research, among others. Notably, the bibliometric results obtained can be markedly influenced by the period of the enrolled literature; consequently, a timely renovation of the literature retrieved is essential for comprehending the research frontier. Bibliometric research on OS and exosome-derived diagnostic biomarkers is scarce.

Hence, we aimed to perform a comprehensive analysis, visualize the correlation between OS and exosomes that can be used as diagnostic biomarkers, and assess the current research status and future trends based on the literature from 1995 to 2023. Additionally, we searched the relevant literature for exosome-derived diagnostic biomarkers used for other tumors to better comprehend the use of exosomes as potential biomarkers.

## 2 Materials and methods

### 2.1 Search methodology

The Web of Science (WoS) core database was searched on October 31, 2023, for all the data used here. Following the outlined procedure, the search algorithm used was: TS = (osteosarcoma) AND TS = (exosomes OR EVs) AND TS = (marker OR biomarkers). Moreover, to further investigate the exosome-derived diagnostic biomarkers, we additionally searched for studies on tumor- and exosome-derived biomarkers from the 2013–2023 period using the TS = (tumor OR cancer) AND TS = (exosome OR EVs) AND TS = (marker OR biomarker) algorithm. After a thorough and repeated screening process, we retained only articles and reviews, eliminated the literature that was not relevant to our research question, constrained the language to plain English, extracted the data, and stored them in the TXT format.

**Figure 1:**
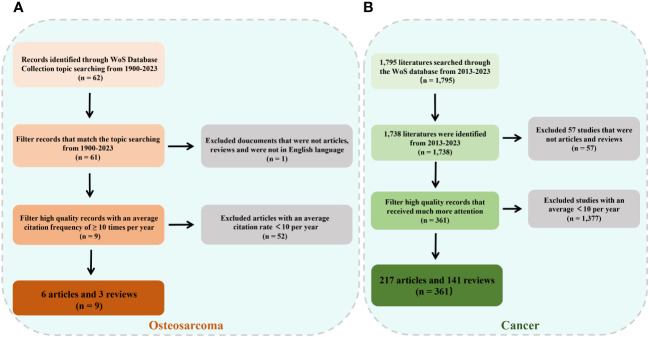
Flowchart of literature screening under bibliometric analysis. **(A)** Flow diagram for osteosarcoma. **(B)** Flow diagram for cancer.

The files were in plain-text format and included complete citations for references to improve the bibliometric analysis and the visualization of our findings. **Figure 1A** (OS) and 1B (cancer throughout the body) present an in-depth overview of the selected information. Furthermore, as a component of the qualitative analysis, we conducted a bibliometric screening of the papers with an average annual citation count of over 10.

This study did not necessitate approval from the Committee on Ethical Medicine.

The final collection of studies on OS from the 1995–2023 period included 61 publications on OS and exosome-derived biomarkers. Moreover, 1,738 documents on tumor- and exosome-derived biomarkers from 2013 to 2023 were found. The extracted data included the journal name, reference type, publication date, author affiliations and names, and abstract.

### 2.2 Analysis and visualization using bibliometric methods

Bibliometrix is a bibliometric software package that was designed by the University of Federico II in Naples, Italy, and is implemented using the R programming language. According to previous research, R4.0.3 bibliometric procedures have been used to automatically assess the publication data (13, 14). This study incorporated data on annual publication counts, publications categorized by country, authors and institutions, journals, co-cited references, and keywords. To assess the quality of the studies, metrics such as the number of published papers, citation counts, and the H-index of the authors were used (14). The H-index of the authors was used to measure the output of researchers in this research field (15). CiteSpace, a scientometric research tool developed by the School of Computing and Intelligence at Drexel University (14), was used for the co-author analysis of the country/author/institution, the analysis of co-citations for a journal or reference, contribution analysis of the keywords, and visualization using grids and overlays, among others.

## 3 Results

### 3.1 Common description of the published literature

**Figure 2:**
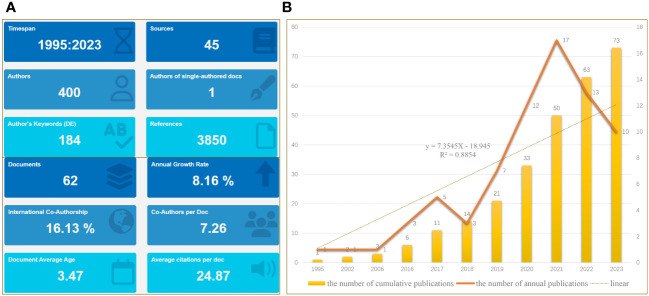
Main information. **(A)** Relevant literature from 1995 to 2023 under bibliometric analysis. **(B)** Graph showing the number of cumulative publications and number of annual publications.

On November 1, 2023, a scientific literature search of the WoS core repository was performed to obtain all relevant published papers related to OS and exosomes/EVs, which serve as biomarkers in clinical diagnosis. Based on the search terms mentioned above, after screening, we initially obtained a total of 62 documents, of which 36 articles (58.06%), 25 reviews (40.32%), and one article were retracted over an almost thirty-year period, from 1995 to 2023 (**Figure 2**). The annual growth rate was 8.16% and the rate of international co-authorship was 16.13%, as shown in **Figure 2A**. Additionally, between 1995 and 2023, although only one article was published in 1995, 2002, and 2006, the annual literature published experienced a rapid upward trend from 2016 to 2021, followed by a rapid decline from to 2021 to 2023 (**Figure 2B**). The evolution trend of the number of cumulative publications fits the fitting curve y = 7.3545x - 18.945 (R² = 0.8854) (**Figure 2B**), which demonstrates that exosomes/EVs have progressively attracted the attention of researchers and may be a long-standing and promising research hotspot.

### 3.2 Nation and district evaluation

**Figure 3:**
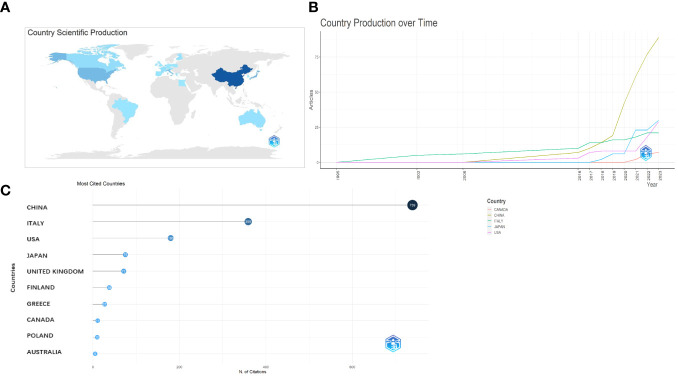
Analysis of the distribution across the world. **(A)** Country scientific production. **(B)** Country production over time. **(C)** Most cited countries.

The corresponding authors were primarily from China, the USA, Australia, Canada, Brazil, France, and Italy (**Figure 3A**). The average paper citations for the top 10 affiliations and leading countries are listed in **Tables 1** and **2**. China and Italy are the main countries in which these studies were published.

**Table 1:** Average article citations for major participating countries in OS diseases.

Country	Total Citation	Average Article Citations
CHINA	739	26.4
ITALY	359	44.90
USA	180	25.70
JAPAN	75	15.00
UNITED KINGDOM	71	71.00
FINLAND	38	19.00
GREECE	27	13.50
CANADA	11	3.70
POLAND	10	10.00
AUTRALIA	5	5.00

**Table 2:** Top 10 affiliations and number of published articles in OS diseases.

Affiliation	Articles
UNIVERSITY OF KANSAS MEDICAL CENTER	7
NATIONAL CANCER CENTER-JAPAN	6
UNIVERSITY OF KANSAS	6
KANAZAWA UNIVERSITY	5
UNIVERSITY OF TEXAS HEALTH SCIENCE CENTER AT SAN ANTONIO	5
SHANGHAI JIAOTONG UNIVERSITY	4
TOKYO MEDICAL UNIVERSITY	4
UNIVERSITY GUELPH	4
UNIVERSITY OF TEXAS SYSTEM	4
SHANGHAI JAIOTONG UNIVERSITY	4

The University of Kansas Medical Center and National Cancer Center, Japan, are the primary affiliations of the authors of these studies.

The nation that published the largest number of documents is depicted in **Figure 3B**. The number of papers associated with China increased quickly and consistently over time, differing from that associated with the USA, which moderately increased (**Figure 3B**). **Table 1** lists the top 10 nations with the highest average article citation rates, along with the individual citation rates in global terms. The average number of article citations from the United Kingdom (71.00%), Italy (44.90%), and China (26.4%) ranked the highest, which is indicative of the superior quality of their articles in this research field (**Table 1**). Additionally, **Figure 3C** shows that the most cited studies were published in China (739) and Italy (359). The top 10 affiliations and article rankings are presented in **Table 2**. The University of Kansas Medical Center, National Cancer Center, Japan, and the University of Kansas are the main institutions associated with research in this field.

### 3.3 Author analysis

**Figure 4:**
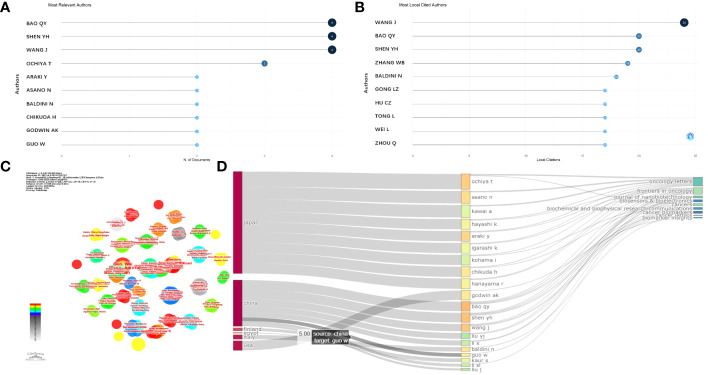
Analysis of authors. **(A)** Most relevant authors. **(B)** Most local cited authors. **(C)** Cluster analysis of cited authors under CiteSpace software. **(D)** Correlation of the states (left), authors (middle), and journals (right) based on the alluvial flow map under R package for osteosarcoma.

Q.Y. Bao (4, 11.33%), followed by Y.H. Shen (4, 11.33%) and J. Wang (3, 12.00%) were the authors with the highest number of article citations in this field, as shown in **Figure 4A**. In addition, all of these authors are those of studies highly cited every year (light blue circles in **Figure 4A**). The local impact of the authors was assessed using the H-index, which was 4, 4, 3, and 2 for Q.Y. Bao, Y.H. Shen, J. Wang, and the other authors (2), respectively, as shown in **Figure 4B**.

Then, the collaboration between authors based on clusters was analyzed, and the connecting lines indicate that the authors were co-authors. The authors Asano, Nanfumi, Chikuda, Hirotaka, Ochiya, and Takahiro (centrality = 0.02) were the core authors with a tremendous mediation centrality (**Figure 4C**). In addition, a cluster evaluation of the cooperative institutes was conducted, as shown in **Figure 4D**. The articles written by Chinese authors were mostly published online in the “Frontiers in Oncology” journal. The authors from the USA mostly published their studies in the journals “Cancer Biomarkers” and “Biomarker Insight,” as exhibited in **Figure 4D**.

### 3.4 Source analysis

**Figure 5:**
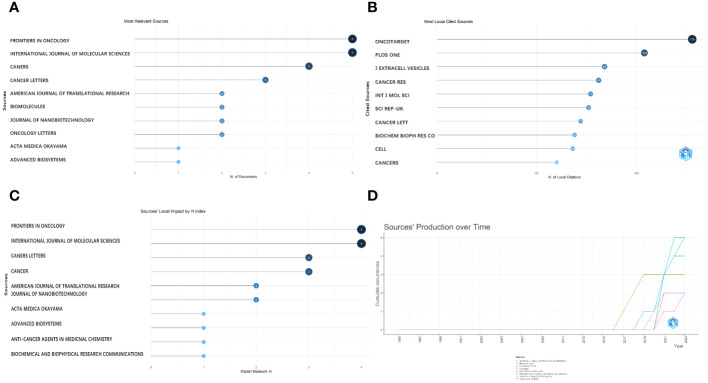
Analysis of journals. **(A)** Most relevant journals. **(B)** Most local cited journals. **(C)** Journals’ local impact by H-index. **(D)** Journals’ production over time.

The most important sources were published in “Frontiers in Oncology,” “International Journal of Molecular Sciences,” “Cancers,” and “Cancer Letters,” which are the main journals in the field of exosomes as OS diagnostic biomarkers (**Figure 5A**). With a high rating in the relevant industry, “Frontiers in Oncology” is a distinguished Journal Citation Reports Division 1 journal, with an impact factor of 4.70. Moreover, most sources cited locally were mainly published in “Oncotarget” and “Plos One”, followed by “Journal of Extracellular Vesicles” and “Cancer Research” (**Figure 5B**). Furthermore, according to **Figure 5C**, “Frontiers in Oncology,” “International Journal of Molecular Sciences,” “Cancer Letters,” and “Cancers” were the main journals in which the main sources that contributed to the local impact according to the H-index were published. Additionally, we found that the related literature began to be published in 2019 (**Figure 5D**).

Choosing an appropriate journal is crucial for scientific researchers, because it allows them to align their research priorities with the journal’s specific aims and scope. This alignment is essential to establish a robust theoretical foundation for OS, particularly when exploring the role of exosomes as biomarkers.

### 3.5 Cited document analysis

**Figure 6:**
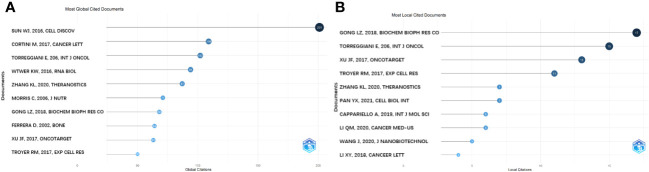
Analysis of documents based on R package. **(A)** Most global cited documents. **(B)** Most local cited documents.

The most globally cited document was “Osteoclast-derived microRNA-containing exosomes selectively inhibit osteoblast activity” (total citation: = 201), which was published in the journal “Cell Discovery” by W.J. Sun, et al., in 2016. The second most globally cited one, “Mesenchymal stroma: Role in osteosarcoma progression” (total citation: = 109) was published in “Cancer Letters” by M. Cortini, et al., in 2017. The third most cited one, “Multimodal transfer of MDR by exosomes in human osteosarcoma” (total citation: = 102) was published in the “International Journal of Oncology” by E. Torreggiani et al., in 2016 (**Figure 6A**). In addition, the most locally cited documents were “Exosomal miR-675 from metastatic osteosarcoma promotes cell migration and invasion by targeting CALN1” (local citation = 17), which was authored by L.Z. Gong and Q.Y. Bao et al. and published in “Biochemical and Biophysical Research Communications” in 2018. This one was followed by “Multimodal transfer of MDR by exosomes in human osteosarcoma” (local citation = 15), which was authored by E. Torreggiani, et al. and published in the “International Journal of Oncology” in 2016. The study titled “Exosomes containing differential expression of microRNA and mRNA in osteosarcoma that can predict response to chemotherapy” (local citation = 13), which was published in 2017 in the journal “Oncotarget” and was authored by J.F. Xu et al., was the third most locally cited document (**Figure 6B**).

### 3.6 Most frequent key words and trend topics

**Figure 7:**
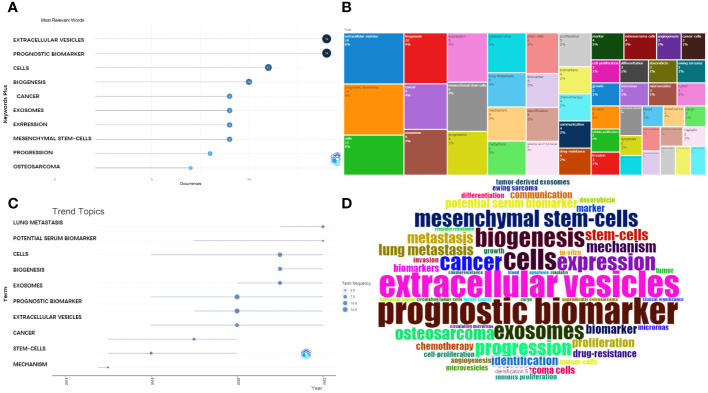
Analysis of keywords. **(A)** Most frequent words. **(B)** Tree map for the relevant words. **(C)** Developmental tendency of topics. **(D)** World-Cloud for the relevant words.

The most relevant key words were: extracellular vesicles, prognostic biomarker, cells, biogenesis, cancer, exosomes, expression, mesenchymal stem cells, progression, and osteosarcoma. These are presented in a plot chart, a word cloud, and a tree map, where the size represents their frequency and importance (**Figures 7A, B, D**).

Furthermore, the main topic subjects were proliferation, mechanism, stem cells, gradually converted into cancer, extracellular vesicles, and exosomes (**Figure 7C**) and, in 2022, lung metastasis and potential serum biomarkers were the most studied subjects. This indicates that a rising number of researchers are exploring the potential connection between the exosomes (or EVs) derived from OS and pulmonary metastasis.

### 3.7 CiteSpace visual evaluation of the authors, institutions, and countries

**Figure 8:**
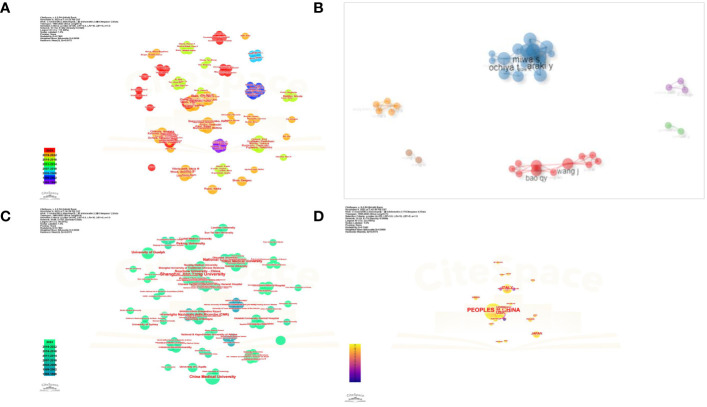
**(A)** Cluster analysis of authors. **(B)** Collaboration network. **(C)** Cluster analysis of institutions. **(D)** Cluster analysis of countries.

A total of 62 documents were included in the analyses of the collaborations between authors, institutions, and countries using CiteSpace visual software. As shown in **Figures 8A, B**, T. Ochiya, Y. Araki, and S. Miwa worked closely with one another. The authors Q.Y. Bao and J. Wang also collaborated closely.

Furthermore, as **Figure 8C** shows, the Shanghai Jiao Tong University, Soochow University, and Fudan University cooperated closely. In addition, the group of the National Cancer Center, Japan, Tokyo Medical University, Okayama University, and the group of Consiglio Nazionale delle Ricerche, University of Bologna, also worked in close collaboration. However, interestingly, the University of Kansas Medical Center reported that most of their articles did not derive from collaborations, which deserves our deep consideration.

The collaboration of groups mainly occurred for groups from China, Japan, the USA, and Italy, which suggests that global teamwork needs to be strengthened (**Figure 8D**).

### 3.8 Co-appearance analysis of the keywords

The frequency and number of keyword occurrences over a given period are valuable for assessing the ongoing and anticipated developments in a particular area of study.

Next, we conducted a co-occurrence network analysis of the keywords using CiteSpace software, and the results were visualized using a visualization diagram, cluster plot, timeline chart, and burstiness graph.

**Figure 9:**
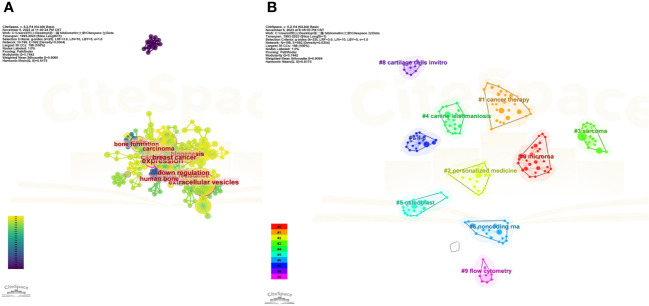
Co-occurrence network analysis of keywords. **(A)** Analysis of VOSviewer. **(B)** Co-occurrence network analysis of keywords based on VOSviewer. In the visualized graph, keywords are divided into 9 clusters with different colors.

**Figure 10:**
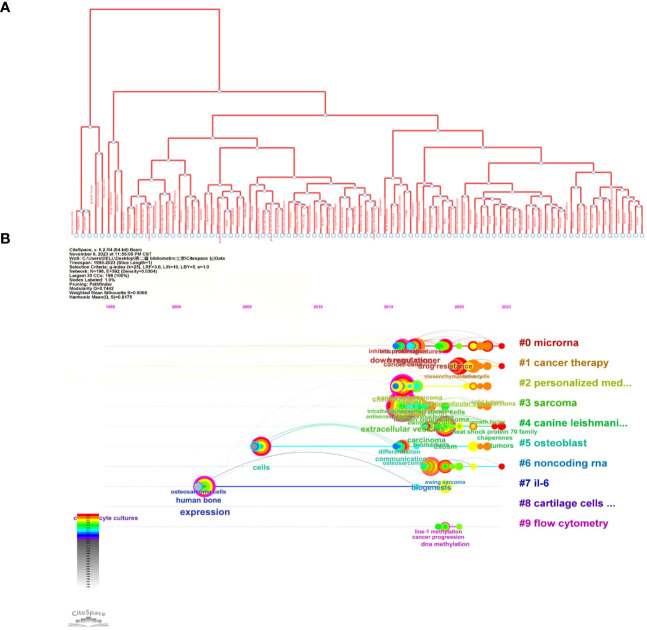
Illustration of the analysis of keywords. **(A)** Trend topics diagram under R package. **(B)** Timeline of keywords based on CiteSpace software.

**Figure 9A** shows the most commonly occurring keywords, which are consistent with the findings presented in **Figure 7**. **Figures 9B**, **10B** are the cluster plot and timeline chart, respectively, which show that nine subject phrases were grouped using the CiteSpace software: microRNA, cancer therapy, personalized medicine, sarcoma, canine leishmaniasis, osteoblast, noncoding RNA, IL-6, cartilage cells *in vitro*, and flow cytometry. The research hotspot gradually shifted toward miRNAs and noncoding RNA, as illustrated in **Figure 10B**.

**Figure 10A** shows the tree map, which illustrates the connection between the keywords and their hierarchical arrangement through hierarchical cluster analysis, which revealed that every subject is composed of a succession of keywords related to “osteosarcoma” and “exosomes.”

### 3.9 Quantitative analysis of the literature included

After applying the Bibliometrix filter with an average citation of ≥ 10 per year, 9 literature pieces (6 articles and 3 reviews) were included (**Table 3**). Next, taken together with the nine studies and 62 documents retrieved using the bibliometric filter, qualitative analysis was conducted not only to clarify the relative research progress on the topic but also to deeply comprehend the intimate relationship between exosomes and the cancer molecular biological mechanisms.

**Table 3:** High-quality literature with an average citation of ≥ 10 times per year in OS diseases.

Author	Title	Journal	Total Citation	Doi
ZHANG KL	EXTRACELLULAR VESICLE-MEDIATED DELIVERY OF MIR-101 INHIBITS LUNG METASTASIS IN OSTEOSARCOMA	THERANOSTICS	87	10.7150/thno.33482
WANG L	PLASMA EXOSOME-DERIVED SENTRIN SUMO-SPECIFIC PROTEASE 1: A PROGNOSTIC BIOMARKER IN PATIENTS WITH OSTEOSARCOMA	FRONTIERS IN ONCOLOGY	30	10.3389/fonc.2021.625109
CORTINI M	MESENCHYMAL STROMA: ROLE IN OSTEOSARCOMA PROGRESSION	CANCER LETTERS	109	10.1016/j.canlet.2017.07.024
TORREGGIANI E	MULTIMODAL TRANSFER OF MDR BY EXOSOMES IN HUMAN OSTEOSARCOMA	INTERNATIONAL JOURNAL OF ONCOLOGY	102	10.3892/ijo.2016.3509
HATTINGER CM	DRUG RESISTANCE IN OSTEOSARCOMA: EMERGING BIOMARKERS, THERAPEUTIC TARGETS AND TREATMENT STRATEGIES	CANCERS	45	10.3390/cancers13122878
PAN YX	CISPLATIN-RESISTANT OSTEOSARCOMA CELL-DERIVED EXOSOMES CONFER CISPLATIN RESISTANCE TO RECIPIENT CELLS IN AN EXOSOMAL CIRC_103801-DEPENDENT MANNER	CELL BIOLOGY INTERNATIONAL	37	10.1002/cbin.11532
GONG LZ	EXOSOMAL MIR-675 FROM METASTATIC OSTEOSARCOMA PROMOTES CELL MIGRATION AND INVASION BY TARGETING CALN1	BIOCHEMICAL AND BIOPHYSICAL RESEARCH COMMUNICATIONS	68	10.1016/j.bbrc.2018.04.016
SUN WJ	OSTEOCLAST-DERIVED MICRORNA-CONTAINING EXOSOMES SELECTIVELY INHIBIT OSTEOBLAST ACTIVITY	CELLDISCOVERY	201	10.1038/celldisc.2016.15
WITWER KW	TOWARD THE PROMISE OF MICRORNAS – ENHANCING REPRODUCIBILITY AND RIGOR IN MICRORNA RESEARCH	RNA BIOLOGY	94	10.1080/15476286.2016.1236172

Liquid biopsy has gained traction in cancer research and is currently undergoing a surge in clinical applications. Liquid biopsies can generally be classified according to the cancer cells they target (17): (i) CTCs, (ii) circulating tumor DNA (ctDNA), and (iii) tumor-derived exosomes and other EVs. Exosomes and other EVs have evolved into versatile platforms with broad and complementary applications. Exosomes contain RNAs, DNAs, proteins, sugar structures, lipids, and metabolites. Moreover, surface molecules on the cytomembrane of exosomes can provide information about the tissue origin, making it possible to enrich the signatures of site-specific cancers.

Furthermore, tumor cells often utilize exosomes, robust components of intercellular communication, to improve their growth (16, 18). Hence, exosome-derived biomarkers show tremendous promise and have been adopted in scientific research and clinics (16, 18–20).

#### 3.9.1 Noncoding RNAs

Structurally, ncRNAs are classified into small ncRNAs (≤ 200 bp; e.g., miRNAs) and long ncRNAs (> 200 bp; e.g., circRNAs or lncRNAs), which are functional RNAs transcribed from DNA but which almost do not encode proteins (21). These ncRNAs serve as a cluster of functional RNAs, are only present in an RNA form, and are indispensable gene expression regulators (21, 22). In the following sections, special RNAs that serve as biomarkers for cancer diagnosis are elaborately reviewed.

##### 3.9.1.1 miRNAs

miRNAs are a category of small RNAs (19–25 nucleotides (nt) of length) that are regulatory RNAs, participating in the stability and translation of the target mRNAs (22). In particular, miRNAs have been linked to biological processes such as cell growth, differentiation, proliferation, metabolism, and apoptosis (22). A dysregulated expression of these miRNAs in numerous solid tumors is important for both cancer initiation and progression (23, 24). Noncoding RNAs (ncRNAs) can be selectively encapsulated into exosomes, indicating that these small molecules may serve as valuable biomarkers (**Table 4**).

**Table 4:** Exosomal ncRNAs as diagnostic biomarkers for osteosarcoma diseases.

ncRNAs	Biomarker Potential	References	Total Citation
MiR-675	A biomarker for osteosarcoma prognosis	H. Zhang et al. (2020) (25)	68
MiR-101	A biomarker for osteosarcoma diagnosis and prognosis	K. Zhang et al. (2020) (26)	87
MiR-130a-3p, miR-195-3P, miR-335-5p, miR-92a-3p, and let-7i-3p	A biomarker for osteosarcoma diagnosis	Ye et al. (2020) (16)	109
Circ0000190	A biomarker for osteosarcoma diagnosis	S. Li et al. (2020) (6)	102

For instance, L.Z. Gong et al. purified exosomes from patient serum and metastatic OS cell lines, and then found that miR-675, which may be a candidate biomarker for OS metastasis, was upregulated, according to the small RNA sequencing and RT-PCR validation results. K.L. Zhang et al. demonstrated that the EVs-miR-101 level was lower in patients with OS than in healthy subjects. Moreover, the EVs-miR-101 expression level was lower in patients with OS with metastases than in those without metastases. Therefore, EVs-miR-101 may be a circulating biomarker for OS. Z.M. Ye et al. collected and purified exosomes from 25 patients with OS and 10 healthy subjects. They found that the miR-335-5p, miR-195-3p, let-7i-3p, miR-130a-3p, and miR-92a-3p expression levels tended to be higher in the patients with OS than in healthy subjects.

Furthermore, S.L. Li et al. obtained 60 OS and 60 control samples and demonstrated that EVs-circ-0000190 could be a biomarker for the diagnosis of OS.

##### 3.9.1.2 circRNAs

CircRNAs are a class of ncRNAs with a covalently closed circular form and without a 5′ tail or a 3′ head. Whole genome sequencing has shown that circRNAs are broadly expressed in various tissues and cell lines (27, 28) and are involved in a wide range of biological processes, such as those involved in tumors.

Carcinogenic circRNAs, such as circTCF25, circMMP9, circ001621, circ0001658, circEPSTI1, and circANKIB1, which are linked to the proliferation, migration, and invasion of cancer cells, were highly expressed in patients with OS. Conversely, carcinostatic circRNAs, such as circLARP4, circ0002052, circROCK1-E3/E4, and circNRIP1, which are related to the biological progression of tumor cells, were expressed at lower levels in patients with OS (29). Moreover, circ_103801 was highly expressed in the OS cell line-derived exosomes and associated with a shorter survival time and chemoresistance in patients with OS (30). Huo et al. revealed that circ_0056285 is upregulated in OS cells and tissues, constituting a prospective biomarker for OS (31).

##### 3.9.1.3 lncRNAs

Q.M. Li performed high-throughput sequencing of 34 OS and 34 paired adjacent tissues, followed by clone formation, a transwell test, a scratch assay, and the establishment of a xenograft model, and showed that lnc00852 was upregulated, suggesting that it can be used as a new tumor biomarker (32).

#### 3.9.2 Proteins

Proteins are the main players in a variety of biological activities, and different types of pathological states are usually associated with protein dysfunction (33).

Exosomes also contain all types of proteins, such as those involved in the transport and fusion of cellular membranes (Rab GTPases, flotillin, and annexins), CD9, CD37, CD53, CD63, Tsg101, and heat shock proteins (Hsp70 and Hsp90), among others, which can affect the parental cell state (34). Exosomal proteins derived from tumor tissues are being developed as promising biomarkers for cancer monitoring and diagnosis. Exosomal proteins have characteristics distinct from those of conventional serological biomarkers and have higher stability, sensitivity, and specificity than those biomarkers (35).

Currently, exosomal proteins are used as diagnostic biomarkers for ovarian, breast, urinary, pancreatic, lung, gastric, thyroid, and colorectal cancer, and melanoma (33). However, few studies on their application in OS have been performed. Wang et al. (36) enrolled 146 patients with OS in a study; IHC and ELISA tests were performed and showed that plasma exosome-derived sentrin SUMO-specific protease 1 (SENP1) may serve as a prognostic indicator in individuals with OS. Furthermore, they found that the plasma exosome-derived SENP1 levels were higher than the plasma-derived SENP1 levels in patients with OS; therefore, the former can be used as a prognostic biomarker.

In summary, scientific research on exosome biomarkers for the diagnosis of OS is still scarce. Studies have shown that the expression of abundant ncRNAs and proteins derived from exosomes can change during the process of OS development. Further investigations are warranted to determine the relationship between the expression levels of exosomes and the regulatory mechanisms, progression, and prognosis of OS.

#### 3.9.3 Others

In addition to the ncRNAs and proteins, DNA, glycans, and lipids carried by exosomes have emerged as prospective indicators of various tumor types (37–42). Nevertheless, relevant research on the diagnosis of OS is scarce and deserves further attention.

### 3.10 Advantages and drawbacks

We conducted a thorough analysis of the applications of exosome-derived diagnostic biomarkers for OS using bibliometric estimation to present the research status and hotspots and predict future research trends in this field for the first time. Moreover, R-bibliometric, VOS, and CiteSpace viewer analyses were conducted to ensure the high objectivity and reliability of the data. We estimated the main tumor and exosomal biomarkers via bibliometric analysis and CiteSpace visualization to deeply and comprehensively understand the developmental trend of exosome-derived diagnostic biomarkers in OS disorders. However, this study has some inherent limitations. First, we obtained these documents only from the WoS database, not selecting data from the Embase, Scopus, PubMed, among others, which may have led to biased data. Second, since the use of exosomal biomarkers for the diagnosis of tumor tissues is an emerging research field, related literature, especially on OS, is scarce. Moreover, the latest online literature published in high-quality journals may be ignored owing to the lower citation counts, as per the citation analysis performed. Third, no relevant keywords with the strongest citation bursts were found based on the CiteSpace results, which demonstrates that the literature on this topic is scarce. Fourth, we did not use uniform criteria when using the CiteSpace software, thus affecting the output data, which may swing slightly under different parameters.

## 4 Discussion

OS, which is a well-known, common, and frequent primary malignant tumor, usually develops in the bone tissue of children and adolescents, accounting for 60% of all bone tumor samples (43). OS has consistently ranked as the second most common cause of death among young individuals with tumors (44, 45).

Currently, therapeutic regimens for preoperative chemotherapy, aggressive surgery, and postoperative chemotherapy are standard procedures (46). Although OS progresses rapidly, no distinct terrible pain or clinical evidence can be found in its early stages. Despite attempts to identify the regulators and molecules associated with the proliferation and metastasis of OS, no substantial breakthroughs in clinical applications related to diagnosis or early detection have been made (47, 48). The limitations of tissue biopsy have been gradually realized and emphasized in the field of precision medicine. In contrast, liquid biopsy, which involves collecting specimens from biological fluids, such as blood and urine, with minimal invasion, has become increasingly popular and has opened up new possibilities for cancer diagnosis and real-time monitoring (49).

So far, ctDNA, CTCs, and exosomes have become the three major branches of liquid biopsy (49, 50). Compared with the other detection techniques, exosome-based liquid biopsy has exhibited tremendous effectiveness.

First, in a 1 mL blood specimen, the concentration of exosomes is approximately 10^6^-10^12^ particles/mL higher than that of CTCs; this way, methods involving the collection of a large amount of urine or blood can be avoided. Second, exosomes have greater natural stability in circulation, even under harsh tumor conditions, as a result of lipid bilayers. In contrast, ctDNAs are rapidly degraded. Third, exosomes, which contain plentiful information from the parental living cells, are more representative than ctDNA, which finitely reflects the information about apoptotic or dead tumor cells (49, 51–53). Tumor-derived exosomes, which are secreted from cancer cells and transport parental tumor cell-derived substances, can serve as tumor diagnostic biomarkers (54).

Exosomes are the most abundant source of miRNAs and also could resistant to degradation from RNA enzymes. Meanwhile, miRNAs also are the ideal molecule for exosome studies (51). Because of the distinct differentiation between tumors and healthy tissues, circRNAs have also been identified as new bioindicators (29). LncRNAs, which are tumor diagnostic indicators, are still in their original state; however, they are also worthy of further research owing to their stability and ease of extraction (55). Exosomal proteins have recently attracted considerable attention from researchers. However, studies on exosomal proteins that serve as diagnostic markers for tumors remain scarce (33). Based on the above bibliometric and CiteSpace software visualization results, we found that the number of studies on this topic remains low, with nearly 61 documents being published from 1995 to 2023. Furthermore, with an average citation rate of ≥ 10 per year, the number of high-quality studies enrolled was only 9. The use of exosomes as diagnostic biomarkers has emerged relatively late; therefore, we concluded that pertinent studies are still in the infant stage.

For a better and deeper understanding of the use of exosomes as biomarkers, we searched the literature on tumor- and exosome-derived biomarkers from 2013 to 2023. Finally, 361 documents with an average citation frequency of ≥ 10 times per year were enrolled. The primary aim of this study was to explore exosome-derived diagnostic indicators for OS, and not for all body tumors; therefore, we did not perform specific bibliometric analyses.

**Figure 11:**
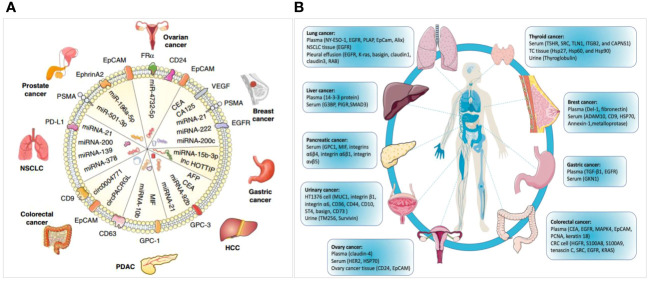
Diagrams for exosome-derived miRNAs and proteins serving as diagnostic biomarkers in cancer. **(A)** miRNAs as diagnostic biomarkers in tumors (6). **(B)** Proteins as diagnostic biomarkers in a variety of cancers (6, 33).

Exosomes can also be used as diagnostic biomarkers for other tumors (**Figures 11A, B**) (6, 33). Hepatocellular carcinoma (HCC) is the most common malignant cancer and the fourth leading cause of tumor-related mortality. Exosomal miR-21 has emerged as a potential molecular marker for the diagnosis of HCC (33). Zhu et al. (56) identified 5 noncoding RNAs (lncHULC, linc01226, circ0073052, circ0080695, and SNORD3B-1) that could serve as promising indicators of HCC. In addition, exosomal proteins such as GPC-3, CEA, alpha-fetoprotein (AFP), albumin, apolipoprotein H, and exosomal miR-122 have been reported as potential diagnostic biomarkers for HCC (56–58). circ0004771, miR21, miR15b, and miR31 expression are elevated in the serum exosomes of individuals with CRC and could be utilized as diagnostic markers for this condition (59, 60). Ma et al. disclosed that the miR21 level was higher in non-small cell lung cancer (NSCLC) samples than in the control samples (61). A high miR-21 expression has also been confirmed in patients with liver, breast, or colorectal cancer (61–63). In addition, Wu et al. indicated that miR-378, miR-139, miR-21, and miR200 were also differently expressed between healthy subjects and patients with NSCLC, making them potential markers for NSCLC (61). Lee et al. found that miR-200c, miR-222, and miR-21 could be used to diagnose BC (63). The level of PSA, which is usually used to detect prostate cancer, is also higher in patients with benign prostatic hyperplasia (64); hence, a specific and sensitive indicator is required for the detection of prostate cancer. Li et al. found that exosomal ephrinA2 may be a potential diagnostic biomarker of this disease (65). Moreover, evidence supporting that the combination of multiple methods, such as exosomal RNA with cfDNA exhibits better advantages exists (66–70). For instance, combining the contents of exosomes (DNA, ncRNAs, proteins, lipids, glycans, among others), cfDNA, and tumor-specific proteins may be useful for developing a diagnostic system, which requires further investigation.

The abovementioned uses of exosomes as diagnostic biomarkers are all based on preclinical research; however, some achievements have been made in translating basic science to a clinical setting. The first commercially available exosome-derived ExoDx™ Prostate (IntelliScore) (EPI) diagnosis test was promoted to evaluate high-grade prostate tumors in 2016 (71, 72). Three exosomal RNA transcripts (ERG, PCA3, and SPDEF) were identified and utilized as prospective signatures for men in the gray zone (a PSA level of 2–10 ng/mL). To date, this diagnostic technique has been used in more than 50,000 patients and has been included in the National Comprehensive Cancer Network guidelines (17). Despite the surplus of studies on the exosomal diagnosis of tumor disorders, only a few have entered the commercial market. While many efforts have been made to use exosomal biomarkers for diagnosing tumors, which is the subject of this article, related studies have expanded to the fields of immune disorders (73, 74), neurodegenerative diseases (75, 76), angiocardiopathy (77), and allograft rejection response (78, 79). Since an elaborate investigation of these topics is beyond the scope of this article, we will not cover them here.

Nevertheless, some unresolved conundrums regarding the role of exosomes still exist. The high heterogeneity of EVs, characterized by their molecular composition and nano size, presents major challenges in isolating and profiling their contents.

So, it is necessary for scientists to precise and accurate the characterization of exosomes. Meanwhile, the analytical approaches to accurately characterize EVs at the single-vesicle level are now under development, and also need to be reliable standardized. There is still a long way to go to realize the clinical transformation of exosomes as diagnostic biomarkers in OS diseases. Therefore, clarifying the mechanism underlying exosome biogenesis could provide a better understanding of these unique diagnostic biomarkers for OS.

## 5 Conclusions

Cancer-secreted exosomes, which contain a large number of substances, such as DNA, mRNA, ncRNAs, and proteins, act as biomarkers for early tumor detection and diagnosis. In recent times, the use of exosomes as diagnostic biomarkers has become an important research topic in this field. However, among the studies on exosome-derived biomarkers for various tumors, the number of those on OS specifically remains low. Diverse molecules, such as proteins, miRNAs, circRNAs, and lncRNAs in exosomes have highlighted potential of diagnostic biomarkers in OS diseases. The current study serves as an important reference for understanding and predicting the future direction of research on exosome-derived diagnostic biomarkers for OS. It also provides guidance for the clinical application of exosome-based liquid biopsy in OS, contributing to advancements in tumor precision medicine.

## Data availability statement

The original contributions presented in the study are included in the article/supplementary material. Further inquiries can be directed to the corresponding author.

## Ethics statement

Ethical approval was not required for the study involving humans in accordance with the local legislation and institutional requirements. Written informed consent to participate in this study was not required from the participants or the participants’ legal guardians/next of kin in accordance with the national legislation and the institutional requirements. The manuscript presents research on animals that do not require ethical approval for their study.

## Author contributions

YP: Writing – review & editing, Writing – original draft, Visualization, Validation, Software, Project administration, Methodology, Investigation, Formal Analysis, Data curation. WG: Writing – review & editing, Supervision, Funding acquisition. YG: Writing – review & editing, Methodology, Investigation. WW: Writing – review & editing, Software, Methodology, Investigation. BW: Writing – review & editing, Formal Analysis, Data curation. FZ: Writing – review & editing, Project administration, Methodology, Formal Analysis. QS: Writing – review & editing, Software, Formal Analysis. JX: Writing – review & editing, Methodology, Data curation. LG: Writing – review & editing, Investigation, Formal Analysis, Data curation. CD: Writing – review & editing, Project administration, Methodology, Formal Analysis. XX: Writing – review & editing, Formal Analysis, Data curation. TR: Writing – review & editing, Supervision, Project administration.
